# Regenerative Effect of Platelet Concentrates in Oral and Craniofacial Regeneration

**DOI:** 10.3389/fcvm.2019.00126

**Published:** 2019-09-03

**Authors:** Faez Saleh Al-Hamed, Mohammed Mahri, Haider Al-Waeli, Jesus Torres, Zahi Badran, Faleh Tamimi

**Affiliations:** ^1^Faculty of Dentistry, McGill University, Montreal, QC, Canada; ^2^Department of Oral and Maxillofacial Surgery, Faculty of Dentistry, Jazan University, Jazan, Saudi Arabia; ^3^Faculty of Dentistry, Universidad Complutense, Madrid, Spain; ^4^Department of Periodontology (CHU/Rmes Inserm U1229/UIC11), Faculty of Dental Surgery, University of Nantes, Nantes, France

**Keywords:** platelet concentrates, clinical applications, growth factors, platelets, oral tissue regeneration

## Abstract

Platelet concentrates (PCs) are biological autologous products derived from the patient's whole blood and consist mainly of supraphysiologic concentration of platelets and growth factors (GFs). These GFs have anti-inflammatory and healing enhancing properties. Overall, PCs seem to enhance bone and soft tissue healing in alveolar ridge augmentation, periodontal surgery, socket preservation, implant surgery, endodontic regeneration, sinus augmentation, bisphosphonate related osteonecrosis of the jaw (BRONJ), osteoradionecrosis, closure of oroantral communication (OAC), and oral ulcers. On the other hand, no effect was reported for gingival recession and guided tissue regeneration (GTR) procedures. Also, PCs could reduce pain and inflammatory complications in temporomandibular disorders (TMDs), oral ulcers, and extraction sockets. However, these effects have been clinically inconsistent across the literature. Differences in study designs and types of PCs used with variable concentration of platelets, GFs, and leucocytes, as well as different application forms and techniques could explain these contradictory results. This study aims to review the clinical applications of PCs in oral and craniofacial tissue regeneration and the role of their molecular components in tissue healing.

## Introduction

Platelet concentrates (PCs) are biological autologous products derived from the patient's whole blood that consist mainly of supraphysiological concentrations of platelets and growth factors (GFs). There are two main types of PCs: platelet-rich plasma (PRP) and platelet rich fibrin (PRF), which can be pure (i.e., P-PRP, P-PRF) or rich in leucocytes (i.e., L-PRP, L-PRF) (1, 2). PCs can also be prepared with or without red blood cells (3). PRP and P-PRF are prepared from anti-coagulated blood, whereas L-PRF is prepared from non-anti-coagulated blood. PC is prepared using gravitational centrifugation techniques, standard cell separators, or autologous selective filtration technique (plateletpheresis) (3). PCs contain high concentrations of growth factors (GFs) and cytokines that play a vital role in the healing of various tissues. PCs have been used alone or as an adjunctive treatment to enhance soft and hard tissue regeneration in dentoalveolar, and maxillofacial surgeries (4–6).

There are four phases of tissue healing: hemostasis phase, inflammatory phase, reparative phase, and remodeling phase. Blood clot serves as a matrix that allows cell movement and proliferation. Platelets are essential for blood clot formation (7). The inflammatory phase starts from the first day to the seventh day after injury (8). In this phase, platelet releases different growth factors that attract inflammatory cells: neutrophils, macrophages and lymphocytes to the injured site. These cells secrete pro-inflammatory cytokines: Tumor necrosis factor alpha (TNF-α), interleukins (IL-1, IL-6, IL-18) that enhance angiogenesis and tissue healing (9, 10). In the reparative phase, mesenchymal stem cells (MSCs) are recruited from the adjacent soft tissue or blood vessels (11). Different growth factors such as bone morphogenic proteins (BMPs) and TGF-β play a role in MSCs recruitment. They enhance bone regeneration as they induce osteoblast differentiation (12). The remodeling phase is characterized by replacement of woven bone with lamellar bone. GFs are also involved in this phase (10, 12).

This study aims to review the clinical applications of PCs in oral and maxillofacial surgery procedures including maxillary sinus augmentation, alveolar ridge augmentation, implant surgery, jaw cysts, periodontal surgery, socket preservation, endodontic surgery, alveolar clefts, cleft lip and palate, oroantral communication (OAC), oral ulcers, osteoradionecrosis, bisphosphonate related osteonecrosis of the jaw (BRONJ), and temporomandibular joint disorders. In addition, the main cellular and molecular components of PCs were discussed with an emphasis on their regenerative properties.

## Types of Platelet Concentrates Used in Oral and Craniofacial Reconstruction

### Platelet-Rich Plasma (PRP)

PRP is the first generation of PCs. It can be prepared using centrifugation or plasmapheresis. The centrifugation can be done in one or two steps (i.e., single or double spin) using various forces (g) and centrifugation times. The centrifugation time ranges from 8 to 30 min (13).The most commonly used centrifugation forces (g) range from 100 to 1,000 g (14, 15), however, some protocols used forces (g) as high as 3,731 g (16).The most common described one step centrifugation protocol is plasma rich in growth factors (*Anitua's PRGF*). In this protocol, plasma is divided into two fractions, the top fraction (platelet poor plasma) and the bottom fraction, including the buffy coat, contains higher concentration of platelets. For the two-step centrifugation protocol, first spin separates the blood in the centrifugation tubes into three layers: red blood cells (RBCs) in the bottom, a buffy coat (BC) rich in platelets and leukocytes in the middle, and platelet poor plasma (PPP) in the top. To produce P-PRP, the PPP layer, and the upper part of the BC layer are transferred to another tube and exposed to a second centrifugation. Then, most of the PPP layer is removed and the platelet pellet concentration in the bottom of the centrifugation tube is resuspended in a small volume of plasma to produce P-PRP. In this protocol, most of leukocytes are discarded. To produce L-PRP, the PPP, the whole BC, and part of RBCs layer are transferred to another tube and centrifuged. The L-PRP consists of the platelet and leukocyte concentration and some RBCs suspended in a small fraction of plasma (1). The increase in platelet count achieved with different PRP methods can range from 2.6 to 7.3 folds, but the two spin protocols the ones that yield higher platelet concentration (13).

PRP can be applied without activation to the recipient site as the exposed collagen or thrombin produced as a result of tissue injury activates platelets (17). However, an activator such as calcium chloride (CaCl_2_), thrombin, collagen, calcium gluconate, lysis by freezing, photoactivation, or a mixture of CaCl_2_ and thrombin is commonlly added before the application. Calcium chloride is the most commonly used activator to counteract the effect of the anticoagulants. The purpose of platelet activation is to enhance the release of GFs from α-granules and to form the gel (17–19) (Table 1).

**Table 1:** Types of platelet concentrates (PCs) (1–3, 19).

**Type of PCs**	**Type of preparation protocol**	**Platelet concentration**	**Leukocytes, + = > 1%**	**Red blood cells, + = > 10%**	**Activation**	**Commercial kits**
PRP	1	A	–	–	I, II, III, IV	Cell separator PRP, Vivostat PRF, Anitua's PRGF
Red PRP	2	B		+		
	3	C				
L-PRP	1	A	+	–	I, II, III, IV	Curasan, Regen, Plateltex, SmartPReP, PCCS, Magellan, Angel, GPS PRP
Red-L-PRP	2	B		+		
	3	C				
PRF	1	A	–	–	I, II, III, IV	Fibrinet PRFM
Red-PRF	2	B		+		
	3	C				
L-PRF	1	A	+	–	I	Choukroun's PRF
Red-L-PRF	2	B		+		
	3	C				

*ISTH classification of PCs: Type of PC preparation protocols: (1) gravitational centrifugation techniques; (2) standard cell separators; and (3) autologous selective filtration technology (plateletpheresis). Platelet concentration: A, platelet count of <900 × 10^3^/μL; B, platelet count of 900–1,700 × 10^3^/μL; C, platelet count of > 1,700 × 10^3^/μL. Activation protocol: I, no activation; II, the use of PRP with activation; III, use of frozen–thawed preparations; IV, photoactivated PRP (not mentioned in ISTH classification)*.

*L-PRF, leukocyte-rich platelet-rich fibrin; L-PRP, leukocyte-rich PRP; PRF, platelet-rich fibrin; Red-L-PRF, red blood cell-rich and leukocyte rich platelet-rich fibrin; Red-L-PRP, red blood cell-rich and leukocyte-rich PRP; Red-PRF, red blood cell-rich platelet-rich fibrin; Red-PRP, red blood cell-rich PRP. + or – defines whether (+) or not (–) there are leukocytes equal or greater than (= >) 1% and/or Red cells equal or greater than (= >) 10% of the total cell counts*.

### Platelet-Rich Fibrin (PRF)

PRF is the second generation of PCs. L-PRF is more commonly used than P-PRF. It can be prepared with a simple inexpensive process without the addition of anticoagulants by using a single spin protocol [3,000 rpm (~700 g) for 10 min] that results in 1–2 folds increase in platelet count (20). The slow polymerization during PRF preparation generates a fibrin network that enhances cell migration and proliferation. In addition, this network acts as a reservoir of platelets, leukocytes, and GFs (21). L-PRF forms a gel that can be applied directly to the surgical site or can be compressed with a special kit to form a membrane that used to cover bone grafts in different augmentation procedures (22).

P-PRF can be prepared only by one method (*Fibrinet PRFM kit*) using two spin protocol. Blood is collected in specified tubes containing tri-sodium citrate and a separator gel, and then centrifuged at 1,100 rpm for 6 min. This separates the blood into three layers: RBCs, BC, and PPP. The BC and the PPP layers are collected and activated with CaCl_2_, followed by second centrifugation at 4,500 rpm for 15 min. Then, platelet rich fibrin matrix (PRFM) clot can be collected and applied to the surgical site (23) (Table 1).

PCs consist of different concentration of platelets, white blood cells, plasma proteins, and GFs. Each component may affect tissue regeneration.

### Platelets

Platelets are ~2.5 μm long cell fragments derived from bone marrow megakaryocytes (24). The normal blood platelet count in healthy individuals ranges from 150,000 to 450,000 platelets/μl. Although platelets lack nuclei, they contain other organelles including mitochondria, microtubules, α-granules, dense granules, and lysosomes (25).

Platelet ultrastructure can be divided into four zones: the peripheral zone, the sole-gel zone, the organelle zone, and the membrane system. The peripheral zone contains the plasma membrane, a smooth membrane rich in glycocalyx [glycoprotein (GP)], lipid bilayer area, and actin filaments. The glycocalyx is essential for platelet adhesion to subendothelial cells, platelet activation, and aggregation (25). The sole gel zone consists of actin microfilaments, a matrix in which all the platelet organelles are suspended, and microtubules (25). The organelle zone contains the secretory organelles: α-granules, dense-granules and lysosomes. The α-granules are the most abundant platelet organelles, each platelet has 50–80 α-granules. The α-granules are round or oval in shape, and 200 to 500 nm in diameter. They contain adhesive proteins (von Willebrand factor (VWF), fibrinogen, and thrombospondin), GFs, microbicidal proteins (thymosin-β4, thrombocidins 1 and 2), immune mediators (complement C3 and C4 precursors), as well as coagulant and fibrinolytic proteins (Factors V, IX, XIII, antithrombin, and plasminogen) (26). The dense granules are smaller than the α-granules. Each platelet contains 3–5 dense-granules. Dense granules contain adenosine triphosphate (ATP), adenosine diphosphate (ADP), serotonin, histamine, polyphosphate, pyrophosphate, calcium, magnesium and potassium. Lysosomes are spherical and each platelet contains <3 lysosomes that contain degrading enzymes (26). The membrane system, besides the plasma membrane, contains the Golgi apparatus, the canalicular system, dense tubules, and the endoplasmic reticulum (27).

The primary function of platelet is to enhance hemostasis through four steps: platelet adhesion, activation, secretion and aggregation. Also, platelets play a vital role in inflammation, tissue healing, and antimicrobial host defense (27).

## Regenerative Properties of Platelet Granules

### α-granules

Platelet α-granules store GFs, adhesion proteins, coagulation and fibrinolytic proteins that are secreted upon activation. These bioactive molecules have bone regenerative properties as they enhance osteogenesis and angiogenesis (28–30). GFs include transforming growth factor-beta (TGF-β), fibroblast growth factors (FGF), vascular endothelial growth factor (VEGF), insulin-like growth factor (IGF), platelet derived growth factors AB (PDGF-AB, PDGF-BB), and brain-derived neurotrophic factor (BDNF) (31). GFs play a role in chemotaxis, proliferation, cell differentiation, the formation of extracellular matrix, osteogenesis, and prevention of bone resorption (29, 30, 32) (Table 2). The clinical applications of GFs are limited due to protein instability, short duration of action, high costs, and they required high doses. This is why PCs are very practical alternatives to deliver GFs (55).

**Table 2:** Main components of platelets and plasma proteins and their effect on bone healing.

**Source**	**Effect on tissue regeneration**	**References**
**PLATELETS**		
**α-Granules**		
IGF	Enhances bone growth, BMD, bone mass and implant osseointegration	(28, 33)
BMP	Plays a role in osteoblast and chondrocyte differentiation and bone regeneration	(29)
PDGF	Improves bone healing and implant osseointegration	(33)
TGF-β	Enhances bone regeneration	(30)
VEGF	Promotes angiogenesis and endochondral ossification	(34, 35)
FGF	Regulates osteogenesis, chondrogenesis, and bone mineral homeostasis	(32)
BDNF	Upregulated in granulation tissue of fractured bone and this suggest its involvement in angiogenesis	(36)
IL-1	Regulates bone metabolism	(37)
IL-6	Modulates inflammation and induces bone resorption	(38)
IL-8	Induces mesenchymal stem cell migration	(39)
**Dense granules**		
Calcium	Increased cytoplasmic Ca^2+^ concentration may induce osteoblast apoptosis	(40)
ATP	Increases bone mineralization at low concentration, but decreases bone mineralization at high concentration	(41)
Serotonin	Reduces bone mineral density and inhibits osteoblast differentiation and proliferation. Inhibits FXIII that mediate the assembly of plasma fibronectin in cell cultures	(42, 43)
Polyphosphate	Inhibits mineralization at low concentration, whereas enhances mineralization at higher concentration in cell cultures	(44, 45)
**BLOOD PLASMA**		
**Albumin**		
	Enhances bone remodeling when used as adjunctive to bone graft material	(46)
**Globulins**		
β-Globulins	Fibronectin, a β-globulin derived protein, enhances re-epithelization and extracellular matrix formation (*in vitro*). Fibronectin combined with β-TCP was slightly better that β-TCP alone in bone regeneration in a rat bone defect model	(47, 48)
**Coagulation proteins**		
Thrombin	Enhances cell migration, osteoblast function, and bone repair	(49)
Factor VIII	Mice lacking F-VIII showed reduced bone healing scores	(50)
Plasminogen	Enhances angiogenesis	(51)
Factor XIII	In osteoblast cell cultures, the inhibition of FXIII-A transglutaminase, resulted in reduction in fibronectin, collagen matrix assembly and mineralization	(43)
Fibrinogen	Fibrinogen 3D scaffolds enhances bone regeneration and increases TGF-β	(52)
**Complement system**		
C1–C9	Maintains cell proliferation and turnover and enhances angiogenesis and tissue healing	(53)
**Other molecules**		
Glucose	High serum glucose (in diabetic patients) reduces bone mineral density and bone turnover by altering osteoblast function and collagen properties	(54)

*PF4, Platelet factor 4; TGF-β, Transforming growth factor beta; FGF, fibroblast growth factors; VEGF, Vascular endothelial growth factors; IGF, Insulin-like growth factor; PDGF, Platelet derived growth factor; BDNF, Brain-derived neurotrophic factor; TGF-β, Transforming growth factor-beta; BMP, bone morphogenetic proteins; F-XIII, Fibrin stabilizing factor; BMD, bone mineral density; NR, not reported*.

### Dense Granules

Platelet dense granules contain ADP, ATP, serotonin, histamine, polyphosphate, pyrophosphate, calcium, magnesium, and potassium. ATP was found to increase bone mineralization at low concentration but decrease bone mineralization at high concentration (41). On the other hand, polyphosphate at low concentration (10 μM) inhibits matrix mineralization in osteoblast cell culutres (44), whereas at higher concentration (100 μM), it enhances osteoblast fucntion and mineralization (45). Serotonin is the fastest-released molecule from platelet after activation, regardless of the type of activation material (56). It reduces bone mineral density and inhibits osteoblast differentiation and proliferation (42). Also, it inhibits FXIII-A, which mediates the assembly of plasma fibronectin in cell cultures (43).

### Regenerative Properties of Plasma Proteins

Plasma proteins are one of main components of blood plasma. They include albumin, globulins (i.e., fibronectin), coagulation proteins (i.e., fibrinogen, prothrombin, thrombin, III, IV, V, VI, VII, VIII, IX, X, IX, XII, XIII), and the complement system. Albumin was reported to enhance bone healing when used as adjunctive to freeze-dried cancellous bone grafts compared to bone graft alone (46). Fibronectin is an adhesive protein that plays a key role in wound healing: enhancing re-epithelization and extracellular matrix formation (ECM) (47). Fibronectin can interact with different types of cells and cytokines to form the ECM through its specific function domains and binding sites (47). In addition, fibronectin with beta-tricalcium phosphate (β-TCP) improve bone regeneration in rat calvarial bone defects (48). Thrombin was found to control osteoblast function and fracture healing in mice (49). Fibrinogen 3D scaffolds improve bone regeneration by increasing transforming growth factor-beta (TGF-β) (52). In osteoblast cell cultures, the inhibition of FXIII-A transglutaminase reduces fibronectin, collagen matrix assembly and mineralization (43). The complement system maintains cell proliferation, cell turnover, and enhances angiogenesis and tissue regeneration (53) (Table 2).

### Leukocytes

PCs may contain leukocytes depending on the preparation protocol. The leukocyte count in whole blood ranges from 4.5 to 11.0 × 10^9^/L. In PCs, leukocyte counts range from 0/L in platelet poor plasma (PPP) to 35.8 × 10^9^/L in L-PRP (57, 58). Leukocytes could enhance or impair tissue healing depending on their environment (58). Neutrophils are the primary white blood cells that migrate to the injured tissue and promote phagocytosis of dead tissue and microbes (59). Monocytes enhance host defense and promote arterogenesis (60). Lymphocytes are not required at the early stages of wound tissue healing, but an innate cellular immune response is required for tissue repair (61). Some studies proposed that leukocytes stimulate the healing process in damaged tissue as they secrete GFs, and simultaneously suppress the growth of bacteria, thus reducing the infection (62–65). However, there is no evidence support the antimicrobial effects of leucocyte- rich PCs (66). Furthermore, other reports showed a positive correlation between the total number of leukocytes in PRP and increased levels of pro-inflammatory cytokines and reactive oxygen species (ROS) released by neutrophils in damaged tissue indicating that high concentration of leukocytes in PRP may inhibit the healing process, and thus the presence of leukocytes should be controlled (67).

### Red Blood Cells (RBCs)

Erythrocytes are the most common cell type of blood cells that carry oxygen to all body tissues. PCs can be prepared with or without erythrocytes. However, many researchers do not pay attention to erythrocyte found in PC and their possible role on tissue regeneration and repair. In addition, there is a lack of clinical and *in vivo* studies assessing the effect of RBCs in tissue regeneration. Thus, the role of erythrocytes on tissue regeneration is unknown. Only an *in vitro* study done by Braun et al. (68) showed that RBC rich-PRP resulted in synoviocyte cell death and thus RBCs may have deleterious effect on cartridge regeneration (68).

### Clinical Applications

**Figure 1:**
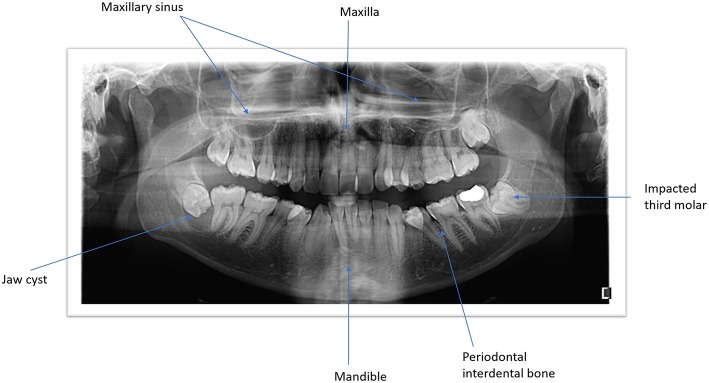
Panoramic view of both jaws illustrating the anatomic structures and the pathological conditions.

**Figure 2:**
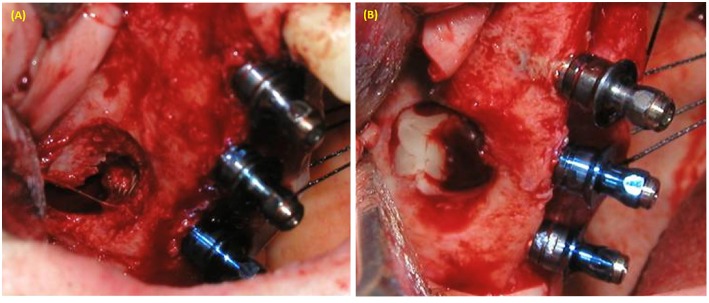
Maxillary sinus augmentation using lateral window approach. **(A)** Perforation of sinus membrane during lateral window approach in sinus lifting with simultaneous implant placement. **(B)** PRP was used to cover the perforated sinus membrane.

**Figure 3:**
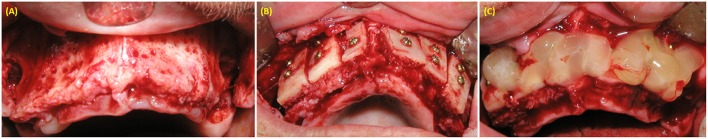
Alveolar maxillary width reconstruction using allograft cortico-cancellous block. **(A)** exposure of alveolar ridge. **(B)** Fixation of bone graft with fixation screws. **(C)** PRP covering the bone graft material.

**Figure 4:**
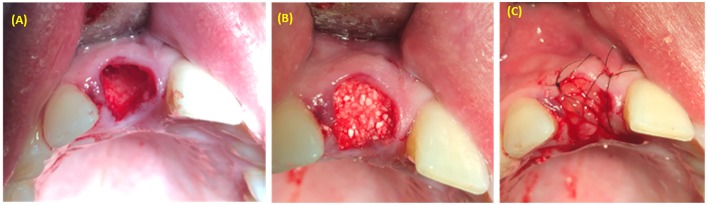
Alveolar socket preservation using bone graft covered with PRP. **(A)** Extraction socket. **(B)** Bone graft applied inside the extraction socket. **(C)** PRP covered bone graft material.

**Figure 5:**
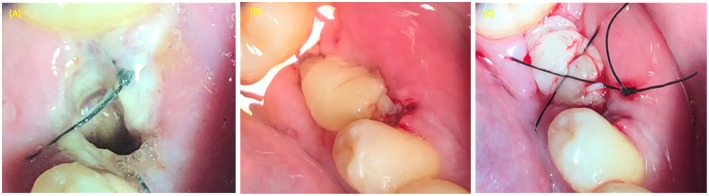
PRP use in treatment of alveolar osteitis (inflammation of alveolar socket). **(A)** Alveolar socket. **(B)** PRP applied in the extraction socket. **(C)** Figure 8 suture type used to stabilize the PRP in the alveolar socket.

**Figure 6:**
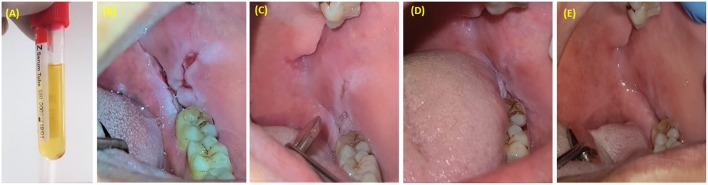
PRP injection as an adjunctive treatment with corticosteroids for resistant oral pemphigus vulgaris. **(A)** 1.5 ml of PRP was injected once a week for 3 weeks. **(B)** Pemphigus vulgaris lesion located posterior to mandibular third molar. **(C)** One week after last injection. **(D)** Six weeks after last injection. **(E)** Six months after last injection.

PCs have been used alone or as an adjunctive treatment to enhance soft and hard tissue regeneration in several oral and maxillofacial interventions such as sinus augmentation, implant surgery, alveolar ridge augmentation, socket preservation, periodontal surgery, endodontic surgery, jaw cysts, oral ulcers, alveolar clefts, jaw osteonecrosis, Oroantral communication, and temporomandibular disorders. Underneath we discuss each one of those interventions in details (Table 3). Figures 1–6 showing some clinical applications of PCs in oral and craniofacial tissue regeneration.

**Table 3:** Clinical applications of platelet concentrates (PCs) in oral tissue regeneration.

**Material**	**Comparisons**	**Effect of PCs**	**References**
**Maxillary sinus augmentation**			
PRF	PRF alone, PRF vs. allograft, PRF vs. xenograft,	PRF alone or combined with allografts accelerate bone healing, although it does not affect the maturation of xenograft	(69)
	PRF vs. collagen membrane	similar new bone formation was reported in both groups	(70)
PRP	PRP with xenograft vs. xenograft	Increased volume of newly formed bone in PRP/xenograft combination	(71)
	PRP with autogenous bone graft or bone substitute	PRP does not improve implant success	(72)
	PRP with bone graft vs. bone graft	PRP did not add beneficial difference to the percentage of newly formed bone or to the implant survival	(73)
**Alveolar ridge resorption**			
PRF	PRF with Ti-Mesh vs. Ti-Mesh	PRF prevents mesh exposure and bone resorption	(74)
	PRF/allograft vs. allograft	PRF increase alveolar ridge width, and the percentages of vital bone	(75)
	PRF/autogenous bone vs. autogenous bone	Adding PRF increases bone width and decreases bone resorption	(75)
PRP	PRGF alone or combined with bone grafts	Enhances soft tissue healing and reduces inflammatory complications	(76)
**Periodontal bone defects**			
PRP	PRP/ bone graft vs. bone graft	PRP as an adjunctive material enhances bone regeneration, reduces pocket depth and enhances clinical attachment level	(77)
	PRP/GTR vs. GTR	No beneficial effect was found when used with GTR	(78)
PRF	PRF alone vs. PRF/ open flap debridement (OFD)	PRF enhance bone regeneration when combined with OFD, whereas it did improve the outcomes with GTR	(78)
**Gingival recession**			
PRF	PRF vs. connective tissue graft	No evidence supports the use of PCs in the treatment of gingival recession	(79)
**Alveolar socket**			
L-PRF	L-PRF vs. blood clot	L-PRF enhanced the preservation of the alveolar width and reduced buccal bone resorption	(80)
L-PRF	L-PRF vs. PRP	L-PRF resulted in better preservation of alveolar height and width compared to PRP	(81)
PRF, PRP	PRF or PRP vs. empty socket	PCs reduce alveolar osteitis compared to empty socket	(82, 83)
PRP, PRF	PRF or PRP vs. empty socket	PCs showed inconsistent results in hard tissue regeneration	(4, 5)
PRF	PRF/bone graft/ bone graft or empty defect	PRF enhances alveolar ridge preservation	(84)
**Implant surgery**			
PRF	PRF/bone graft/ bone graft or empty defect	PRF improves the early phases of osseointegration	(84)
PRF	PRF vs. no graft	PRF enhances implant stability and reduces marginal bone loss	(80)
PRP	PRP around dental implant	Inconclusive results due to lack of studies	(85)
PRF	PRF vs. no graft	PRF does not improve bone healing	(86)
PRF	PRF vs. flap surgery (in peri-implantitis)	PRF improves clinical outcomes	(87)
**Endodontic diseases**			
PRF, PRP	PRF or PRP	PCs enhance bone regeneration, root development, and regaining of pulp vitality, although the level of evidence was weak	(88)
PRF, PRP	PRF or PRP	PCs enhance root development	(89)
**Jaw cysts**			
PRP	PRP/bone graft vs. bone graft	PRP accelerates bone healing	(90, 91)
PRP	PRP vs. empty defect	PRP did not improve bone healing	(92)
**Cleft palate**			
PRP	PRP vs. surgical closure alone	PCs enhance soft tissue closure of complete cleft palate and reduce the incidence of oronasal fistula	(93)
	PRGF with bone graft	Mixing the PRGF with bone grafts resulted in complete closure of 90.9% cases with recurrent cleft palate fistulas	(94)
**Alveolar clefts**			
PRP	PRP with iliac bone graft vs. bone graft	The combination of iliac graft and PRP reduce bone resorption compared to iliac graft	(95, 96)
PRF	PRF with iliac bone graft vs. bone graft	adding PRF to iliac bone grafts, did not enhance maxillary alveolar bone thickness, height, and density or the percentage of newly formed bone	(97, 98)
**BRONJ**			
PRF	PRF with surgical debridement	PRF has been found to enhance soft tissue healing and reduce pain of surgically debrided BRONJ cases	(99)
PRP	PRP with laser	Laser assessed surgery and PRP application enhance tissue healing of BRONJ cases	(100)
L-PRF	L-PRF with BMP-2	The combination of BMP-2 and L-PRF accelerate healing compared to L-PRF alone	(101)
**Osteoradionecrosis**			
L-PRF	L-PRF	Application of PCs combined with surgical debridement improve tissue healing	(102)
PRP	PRP gel	PRP enhance bone regeneration of mandibular bone defect	(103)
**Oroantral communication (OAC)**			
PRF	PRF vs. buccal advancement flap	PRF clots provides successful results and reduces pain and swelling compared to buccal advancement flap.	(104)
PRF	PRF	PRF membrane could be used to manage OAC of size ≤ 5 mm	(105)
**Oral ulcers**			
PRP	PRP	Application of PCs in autoimmune derived ulcers accelerate tissue healing and reduce pain and discomfort during mastication	(106, 107)
PRF	PRF	PRF membrane could improve tissue healing after excision of oral mucosal lesions such as leukoplakia and lichen planus	(108)
**TMDs**			
PRP	PRP vs. Hyaluronic acid or saline injection	PCs injection reduces pain and improves mouth opening in patients with TMDs	(109)

*PRF, platelet rich fibrin; PRP, platelet rich plasma; PRGF, plasma rich in growth factors; PCs, platelet concentrates; BRONJ, Bisphosphonate related osteonecrosis of the jaw; TMDs, Temporomandibular disorders; BMPs, bone morphogenic proteins*.

### Sinus Augmentation

Sinus augmentation is a surgical procedure aims to restore the resorbed bone in the posterior maxilla caused by tooth loss and is indicated in cases with no enough bone height to accommodate dental implants. PRF has been used as sole filling material or combined with bone graft materials in sinus augmentation. The use of PRF as a sole filling material with simultaneous implant placement showed successful results, although there was no control group and these findings could be similar to the use of implant as a tent in which blood clot filled the gap around the dental implant. Furthermore, PRF reduced the healing time when combined with demineralized freeze-dried bone allograft. However, it does not affect the maturation of deproteinized bovine bone (69). In addition, PRF can be used as a membrane to cover the lateral window approach in sinus augmentation. The use of PRF or collagen as membranes in sinus augmentation with xenografts showed similar results in terms of percentages of new bone formation and the amount residual bone (70). The addition of PRP to anorganic bovine bone (ABB) increased the volume of newly formed bone and improved the osteoconductive properties of ABB, although it did not affect the implant success compared to ABB alone (71). However, in a systematic review, assessed the effect of PRP combined with bone grafts in sinus augmentation, showed no differences in clinical outcomes including implant success and complications (72). Also, the use of PCs as an adjunctive treatment for maxillary sinus elevation was not supported by other studies (73). Therefore, further standardized randomized clinical trials (RCTs) are requried.

### Alveolar Ridge Augmentation

Different augmentation techniques such as ridge augmentation, guided bone regeneration (GBR), and ridge splitting and expansion can be used to manage alveolar ridge resorption. Different bone grafts (autografts, allograft, xenografts, alloplasts) with or without biological materials such as PCs are used for alveolar ridge augmentation. In alveolar ridge augmentation with the titanium mesh (Ti-mesh), it was found that covering the mesh with PRP prevents the mesh exposure and bone resorption (74). PRP was found to significantly increase alveolar ridge width and the percentage of vital bone achieved with cancellous allograft (75). In addition, covering the autogenous bone blocks in anterior maxillary augmentation with PRF increased bone width and decreased bone resorption (110). On the other hand, limited evidence was found regarding the effect of PRGF on new bone formation when combined with other bone substitutes, but it also shown some benefits on soft tissue healing, pain, and swelling (76). However, due to limited studies, further research is required.

### Periodontal Surgery

PCs have been used alone or as an adjunctive material in the treatment of intra-bony periodontal defects. Overall, PRP as an adjunctive material significantly enhanced periodontal bone healing (77). PRP or PRF can reduce pocket depth and enhance clinical attachment level (77). Moreover, the use of PRP combined with bone grafts was found to enhance periodontal bone healing, whereas, no beneficial effect was found when used with guided tissue regeneration (GTR) (78). PRF was effective when used alone or combined with open flap debridement, although, there was a lack of evidence regarding its role when combined with bone grafts or GTR (78).

Regarding the efficacy of PCs in periodontal plastic surgery, studies showed that PRF membrane did not regenerate gingival recession, clinical attachment level, or the width of keratinized mucosa compared to other biomaterials such as connective tissue grafts (111).

### Socket Preservation

Platelet concentrates have been used to accelerate healing and regenerate bone in socket preservation procedures. L-PRF reduced the healing time and bone resorption when applied on the extracted tooth socket (80). Moreover, L-PRF was reported to enhance alveolar socket preservation in height and width compared to PRP (81). In addition, PCs reduce alveolar osteitis compared to empty socket after third molar extraction (82, 83). However, PCs have a beneficial effect in accelerating wound healing and reducing postoperative symptoms; some studies showed conflicting results in hard tissue regeneration (4, 5). Overall, there was a moderate evidence to support the use of PCs in socket preservation (84).

### Implant Surgery

Platelet concentrates have been used to enhance bone regeneration prior to implant placement or to treat bone defects in cases of peri-implantitis. Overall, there is moderate evidence to support the use of PCs in the early phases of osseointegration (84). Both PRF and PRP have been found to enhance bone regeneration and reduce marginal bone loss around dental implants (80, 112). However, the application of PRF during immediate implantation does not seem to improve implant stability or bone healing (86). In cases of peri-implantitis, PRF application after surgical debridement was found to reduce peri-implant pocket depth and to increase keratinized mucosa compared to conventional flap surgery only (87).

### Regenerative Endodontics

PCs have been used in different root canal procedures including apexification, apexogenesis, pulpotomy, and endodontic apical surgery. PCs have been found to enhance peri apical bone regeneration, root development, and pulp vitality (88). In addition, a comprehensive systematic review of clinical evidence, showed that application of PCs are successful procedures in the treatment of immature teeth, although the level of evidence was weak (only 5 RCTs and 21 case reports were included), and thus further well-designed RCTs with longer follow-up are required (89).

### Jaw Cysts

Jaw cysts are pathological cavities formed within the jaw bones or soft tissues, that are usually lined by epithelial layer. Radicular cysts are the most common inflammatory cysts that are usually located around the apexes of necrotic teeth. The standard treatment for inflammatory jaw cysts is enucleation (113). Fewer reports assessed PCs application in bone regeneration after cyst enucleation. Filling the cyst cavity with bone graft and PRP was found to accelerate bone healing compared to bone graft only (90). In addition in a pilot study consisted of 10 cystic lesions, PRP was found to significantly accelerate bone healing after 6 months of follow-up (91). However, in another study, PRP did not show any significant acceleration in bone healing after 6 months of treatment compared to the control group (empty defect) (92). Thus, due to limited evidence and absence of RCTs, further standardized studies are required.

### Cleft Lip and Palate

Cleft lip and plate are congenital defects in maxillary and nasal processes that resulted in cleft lip and/or palate. Surgical closure is the treatment of choice in such patients. PRP was found to enhance soft tissue closure of complete cleft palate and to reduce the incidence of oronasal fistula compared to surgical closure alone. Therefore, it could reduce the need for additional surgical procedures (93). In addition, mixing the PRGF with bone grafts resulted in complete closure of 90.9% cases with recurrent cleft palate fistulas (94). However, due to limited evidence, further standardized studies are required.

### Alveolar Clefts Reconstruction

Alveolar clefts are congenital bone defects in the alveolar bone that affect 75% of cleft lip or cleft lip and palate patients (114). The combination of iliac graft and PRP reduce bone resorption compared to iliac graft alone in patients requiring alveolar cleft augmentation (95, 96). On the other hand, adding PRF to iliac bone grafts, did not benefit maxillary alveolar bone thickness, height, and density or the percentage of newly formed bone (97, 98). However, due to limited evidence, further studies are required.

### Bisphosphonate Related Osteonecrosis of the Jaw (BRONJ)

BRONJ is commonly manifested by exposed necrotic bone that persists for 8 weeks or more in patients with metastasis or osteoporosis under antiresorptive medications such as alendronate. PRF has been found to enhance soft tissue healing and reduce pain of surgically debrided BRONJ cases (99). In addition, Erbium Chromium: Yttrium Scandium Gallium Garnet laser (Er,Cr:YSGG) assessed surgery combined with PRP application enhance healing of BRONJ cases (100). The combination of bone morphogenetic protein 2 (BMP-2) and L-PRF in treating BRONJ cases was found to accelerate healing compared to L-PRF alone (101). However, in a systematic review summarizing the combination protocol (surgery and PCs), concluded that there is no sufficient data to support this protocol and thus further studies are required (115).

### Osteoradionecrosis

Osteoradionecrosis of the jaws is defined as bone death caused by radiation therapy of head and neck cancer. PCs have been found to be effective in treating patient with osteoradionecrosis (103). The application of L-PRF combined with surgical debridement using piezosurgery in managing cases of osteoradionecrosis, resulted in complete healing of 67% of cases within 1-year follow-up (102). This promising application could reduce the need for bone resection in such cases, although due to the limited evidence, further research is required.

### Oroantral Communication

Oroantral communication (OAC) is an abnormal communication between the oral cavity and the maxillary sinus that could occur due to pathological conditions or during extraction of maxillary posterior teeth. The treatment of OAC depends on the size of the communication and it is usually achieved by buccal advancement flaps. PRF clots enhance wound closure and reduce pain and swelling compared to buccal advancement flap (104). In addition, PRF membrane could be used to manage OAC of size ≤ 5 mm (105). However, further standardized studies are required to shed the light in such promised application.

### Oral Ulcers

Pemphigus vulgaris is an autoimmune disease that affects the skin and mucous membranes. It manifests in oral cavity as painful erosive lesions (ulcers) that are usually treated with corticosteroids (116). In such cases, the repeated injections of PRP accelerate tissue healing and reduce pain and discomfort during mastication (106). Oral ulcers can also occur after bone marrow allogeneic transplants resulting in graft vs. host disease (GvHD). Application of PCs gel in those patients reduces pain, enhances mastication, and accelerates healing (107). In addition, PRF membrane could improve tissue healing after excision of oral mucosal lesions such as leukoplakia and lichen planus (108). PCs seem to be promising materials for managing oral ulcers, however, due to limited studies, further research is required.

### Temporomandibular Disorders (TMD)

TMDs are diseases of multifactorial origin that affect the temporomandibular joint articular surfaces as well as the surrounding masticatory muscles (117). PRP injection has been used to reduce pain and to improve mouth opening in patients with TMDs. In a systematic review, PRP injection was found to reduce pain and improve jaw movements in 4 out of 6 studies compared to hyaluronic acid or saline injections (109).

## Discussion

PCs have been used extensively in oral and craniofacial interventions for hard tissue regeneration. Overall, PCs seem to have a beneficial role on bone regeneration in different clinical procedures, such as socket preservation, implant surgery, sinus augmentation, periodontal surgery, osteonecrosis, and alveolar ridge augmentation (71, 75, 78, 80, 90, 95, 96, 100, 112), although, there was some inconsistent findings. The regenerative potential of PCs is attributed to their contents of bioactive molecules, specifically GFs known to enhance osteogenesis, angiogenesis and tissue regeneration (28–30). However, due to limited studies, further research is required.

PCs seem to improve soft tissue healing in socket preservation (81), periodontal surgery (77), oral ulcers (106), oroantral communication (105), osteonecrosis (100), and alveolar ridge augmentation (74, 75). The improvement on soft tissue closure obtained by PCs could indirectly enhance bone regeneration in different surgical procedures.

In addition, PCs could reduce inflammatory complications such as pain and swelling and could improve mouth opening and masticatory function in patients with in temporomandibular disorders (TMDs) (109, 118), oral ulcers (106), jaw osteonecrosis, and extraction sockets (4). This beneficial role could be attributed to their content of GFs that have an essential role in inflammation, cell movement and metabolism (119). In addition, PCs may have immunomodulatory effects; PC can induce considerable changes in the level of proinflammatory mediators such as an increased level of lipoxin A4 (LXA_4_) and thus suggests that PCs could prohibit cytokine secretion, reduce inflammation and promote tissue healing (120).

However, the effect of PCs on bone regeneration has been clinically inconsistent across the literature (4, 76). Differences in study designs and types of PCs used with variable concentration of platelets, growth factors, and leucocytes, as well as different application forms and techniques could explain these contradictory results (6). Also, the centrifuge characteristics and different centrifugation protocols could affect the cells, growth factors and fibrin architecture of PCs and thus any modification of the original protocols should be investigated separately in order to avoid confusion and inaccurate results (121). In addition, a recent study suggested the possible negative effect of platelet dense granule contents including serotonin in bone regeneration (23). Serotonin inhibits osteogenic activity and osteoblastic differentiation and proliferation (42, 122, 123). Therefore, PCs preparation protocols require standardization.

PRP and PRF are the main types of PCs that applied in different clinical procedures. PRP is the only PCs that can be injected inside the temporomandibular joint (TMJ) spaces in treating patients with TMDs (109, 118). PRF membrane is used for closure of oroantral communication (105). Both materials could be used for other clinical interventions. Their main challenges are the stability in the application site and the need for an optimized delivery system that allow gradual release of growth factors (124).

## Conclusion

Platelet concentrate (PC) are biological blood-derived products that are rich in platelets and GFs. Overall, PCs seem to enhance bone and soft tissue healing, reduce pain and swelling in alveolar ridge augmentation, periodontal surgery, sinus augmentation, socket preservation, implant surgery, endodontic regeneration, BRONJ, osteoradionecrosis, closure of oroantral communication (OAC), oral ulcers, and temporomandibular disorders. On the other hand, no effect was reported in gingival recession and guided tissue regeneration (GTR) procedures. However, due to the limited available evidence and the heterogeneity among different studies, further research is required to shed light on these promising biological materials.

## Author Contributions

FA-H wrote the review and participated in design the review. MM participated in writing part of the clinical applications. HA-W revised the manuscript. JT revised the manuscript. ZB and FT designed and revised the manuscript.

### Conflict of Interest Statement

The authors declare that the research was conducted in the absence of any commercial or financial relationships that could be construed as a potential conflict of interest.
